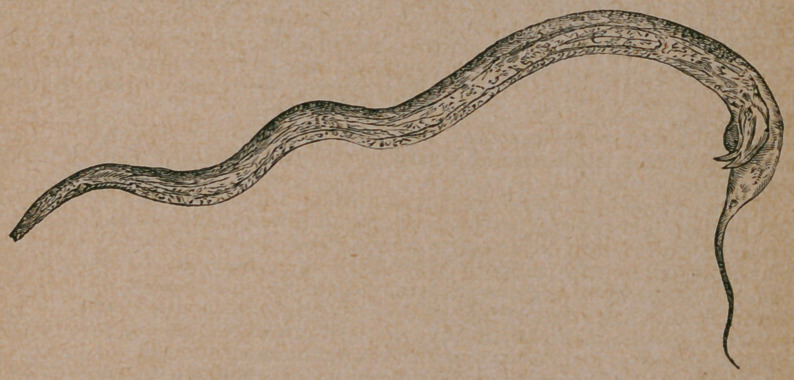# Progress of Veterinary Science

**Published:** 1884-04

**Authors:** 


### P[illegible]gress of Veterinary Science.

THE CAUSE OF "SEEDY TOE."—Prof. J. Wortley Axe, in the *Veterinarian* for January, gives an account of a discovery made by him concerning the above named disease. He declares the cause of it to be a parasite, shown in the illustration, which he found on making a microscopical examination of the debris found in the cavities of the hoof. Prof. Cobbold to whom these organisms were submitted for further examination recognized the importance of the discovery and explained the manner in which the worm destroyed the horny tissue by means of a boring spike at their oval extremity. He named it after the discoverer, *Pelodera Axei.* They obtain entrance to the hoof substance through nail holes and fissures found in "shelly feet."

Prof. Axe states, however, that he has examined carefully some cases of the disease without finding the parasite, but does not therefore conclude that they have no influence in causing the disease. He thinks it advisable to continue the inquiry and requests members of the profession to forward to him for examination portions of the "borings" from the cavity. Those who desire to make the examination themselves may do so by macerating a portion of the debris and afterwards teasing it out on a slide, when it may be viewed with a half-inch objective.

Should the evidence be in favor of a parasitic origin of the disease the treatment will be obvious.

A CASE OF CRAMP IN A COW—SECTION OF LEGAMENTUM PATELLA INTERNUM—RECOVERY.—An anomaly in the position of the patella existed in the left hind joint more than a year in one of my cows, of the Piedmontese race.

It first declared itself in the animal while working by a difficulty in flexing the leg on the thigh, a spasmodic contraction taking place accompanied by a crackling noise in the articulation.

The disorder progressed and one month before the operation it had acquired all the characteristics of cramp in its most severe form. On arising from the lying posture the animal was invariably attacked by the cramp which continued violent for some time so that at last she dreaded to make the least movement of the affected limb. Treatment by the usual method failed to give relief and the only alternative seemed to allow the cow to walk on three legs or remain in the stall

I then determined to operate. Making an incision on the skin of the inner side of the joint, I pushed the knife under the ligament until it reached the skin on the outside. Substituting now a tenetome, I introduced it, edge dogs barking furiously from behind their iron-railed kennels, are doomed to death. These inhabitants of the laboratory, which are marched out day after day in order to be subjected to operations or other experiments, share the space with still more ghastly objects. From all parts of France hampers arrive containg fowls which have died of cholera or some other disease. Here is an enormous basket bound with straw; it contains the body of a pig which has died of fever. A fragment of lung forwarded in a tin box, is from a cow dying of pneumonia. Other goods are still more precious. Since M. Pasteur, two years ago, went to Pauillac to await the arrival of a boat which brought yellow fever patients, he receives now and then from far-off countries a bottle of *vomito negro*. Tubes filled with blood are lying about, and small plates containing drops of blood may be seen everywhere on the work tables. In special stores bottle-like bladders are ranged resembling small liquor bottles. The prick of a pin into one of these bladders would bring death to any man. Inclosed in glass prisons millions and millions of microbes live and multiply." M. Pasteur's views on vivisection are known well enough. His own words on the subject are : "Never should I have the courage to kill a bird for sport, but when it comes to experiments I have never been troubled by the slightest scruple. Science in that case has the right of pleading the sovereignty of the purpose."

THE DEPARTMENT OF VETERINARY MEDICINE IN THE UNIVERSITY OF PENNSYLVANIA.—We are informed that the organization of this department of the University of Pennsylvania has been completed, and the members of the faculty, with the exception of two or three special teachers, appointed. The faculty includes William Pepper, M.D., LL.D., Provost of the University and *ex-officio* President; Rush Duffen Huidekoper, M.D., V.S., Dean, Professor of Internal Pathology, and *pro tempore* Professor of Veterinary Anatomy; James Tyson, M.D., Professor of General Pathology and Morbid Anatomy; Horatio C. Wood, M. D., LL.D., Professor of Materia Medica, Pharmacy,and General Therapeutics; Theodore G. Wormley, M.D., LL. D., Professor of Chemistry and Toxicology; Harrison Allen, M D., Professor of Physiology ; Joseph T. Rothrock, M.D., B.S., Professor of Botany ; Andrew J. Parker, M. D., Ph.D., Professor of Comparative Anatomy and Zoölogy: Robert Meade Smith, M.D., Professor of Comparative Physiology; —— ——, Professor of Surgical Pathology and Obstetrics ; Adolph W. Miller, M.D., Ph.D., Demonstrator of Pharmacy ; Henry F. Formad, M.D., B.S., Demonstrator of Pathology and Morbid Anatomy.

The course of study extends over three years, the year beginning October I and ending on June 15. There quirements for admission to this department are the same as for the Department of Medicine the studies for

*First Year.*—Chemistry, Materia Medica and Pharmacy, Physiology, Histology, Botany, Zoölogy, Veterinary Anatomy and Forging.

*Second Year.*—Medical Chemistry, Physiology, Therapeutics, General Pathology and Morbid Anatomy, Veterinary Anatomy, Surgical Pathology, Internal Pathology and the Contagious Diseases, Botany and Zoölogy.

*Third Year.*—Therapeutics, General Pathology and Morbid Anatomy, Surgical Pathology and Operative Surgery, Internal Pathology and the Contagious Diseases, Sanitary Police, Obstetrics and Zootechnics.

In the second year the student will attend clinics, and will serve as aid in the hospital ; in the third year, he will be placed in charge of sick animals, and be required to prepare clinical reports and make autopsies. He will also make regular visits to breeding and dairy farms and to slaughter-houses, in order to familiarize himself with the race of animals, the economical means employed in their care, and the varieties of butcher-meat.

*Examinations* will be held at the close of each year and at the end of the course. All these examinations must be passed satisfactorily before the student can be registered as a candidate for the degree.

*Graduation.*—Upon completing satisfactorily the full course of study, the student receives the degree of Veterinary Surgeon (V.S.), upon the same conditions as those on which the degree of Doctor of Medicine is conferred.

A handsome ampitheatre and large rooms for dissection and labratory work have been erected, and the stables requisite for hospital use will be finished and occupied before the opening of the department in September, 1884

## Figures and Tables

**Figure f1:**